# The Hidden Tear: Improving Diagnostic Accuracy for Spontaneous Esophageal Perforation in the ED

**DOI:** 10.7759/cureus.65482

**Published:** 2024-07-27

**Authors:** Marwa Morgom, Leena Saeed, Hanna Ali, Hadeel Elhassan

**Affiliations:** 1 Emergency Medicine, Hamad Medical Corporation, Doha, QAT; 2 Internal Medicine, Medical Research Center, Hamad Medical Corporation, Doha, QAT; 3 General Practice, Hamad General Hospital, Doha, QAT

**Keywords:** imaging, chest x ray, imaging modalities, boerhaave's syndrome, esophageal perforation

## Abstract

Esophageal perforation is a serious medical condition characterized by a tear or hole in the muscular layer. This case report details the presentation, diagnosis, and treatment of a patient with missed esophageal perforation at an emergency department. The report highlights treatment options, missed findings from the chest X-ray, and relevant case details. Management primarily depends on prompt detection and intervention through conservative measures or surgical repair. Identifying the issue within the initial hours after presentation can significantly decrease the mortality rate, which can be as high as 30%.

## Introduction

This introduction dives into the dangers of esophageal rupture, a life-threatening condition in which the esophagus tears open due to intense pressure. Boerhaave’s syndrome, as it is called, is distinct from the less severe Mallory-Weiss tear [1].

The symptoms of esophageal rupture can be misleading, making diagnosis difficult. The classic signs of vomiting, chest pain, and air under the skin (Mackler’s triad) are rarely present. Delays in diagnosis and treatment can lead to serious complications like infection and organ failure [1]. A study highlighted the association between esophageal rupture and forceful vomiting, often triggered by large meals and alcohol intake [2].

Emergency physicians face a challenge. Even if a patient does not exhibit all the typical symptoms, the possibility of esophageal rupture cannot be ruled out [1]. Chest X-rays often prove unhelpful, offering only subtle clues. More advanced imaging is crucial for confirming the diagnosis, assessing the tear’s severity, and guiding the best course of treatment [3].

Treatment of esophageal ruptures can be challenging. Such conditions are often treated conservatively; patients are kept NPO, administered antibiotics and proton-pump inhibitor (PPI) intravascularly, given a nasogastric tube for suctioning and decompression, and started on chest drainage if necessary. In addition, close follow-up should be maintained so that the opportunity for surgery is not missed when necessary [3].

Our case explores the challenge of diagnosing esophageal rupture in an 18-year-old with recurrent vomiting. While forceful vomiting is a common cause, other factors and coexisting conditions can complicate the picture. Delays in treatment can be deadly, with mortality rates reaching up to 40%. Without intervention, the risk of death soars to a staggering 90% [4].

## Case presentation

An 18-year-old man with no prior medical history visited the emergency department for recurrent attacks of vomiting aggravated by spicy food and stress related to his exams. The patient mentioned subtle chest and abdominal discomfort. He was tachycardiac and tachypneic at the time of assessment. He denied any recreational medication use or alcohol intake.

Upon examination, the patient was noted to be anxious and dehydrated. He exhibited mildly elevated heart and respiratory rates, but his other vital signs were within normal limits. The patient’s body mass index was 17 kg/m². His recorded vital signs included a temperature of 37°C, blood pressure of 125/70 mmHg, pulse rate of 125 bpm, respiratory rate of 14 breaths per minute, and oxygen saturation of 98% on room air.

Laboratory results revealed no significant findings, and all values for complete blood count, comprehensive metabolic panel, and lipase were within the normal range (Table 1).

**Table 1:** Initial laboratory results.

Group	Detail	Value w/Units	Flags	Normal Range
Blood Chemistry	Bicarbonate	20 mmol/L	LOW	22–29
Blood Chemistry	B-Hydroxybutyrate	4.13 mmol/L	HIGH	0.03–0.30
General Hematology	WBC	13.1 x 10^3^/uL	HIGH	4.0–10.0
Endocrinology	Vitamin D	11 ng/mL	DEFICIENCY	≥ 20 ng/mL Optimum Values
Blood Chemistry	Sodium	135 mmol/L	NORMAL	133–146
Blood Chemistry	Potassium	3.6 mmol/L	NORMAL	3.5–5.3

**Figure 1:**
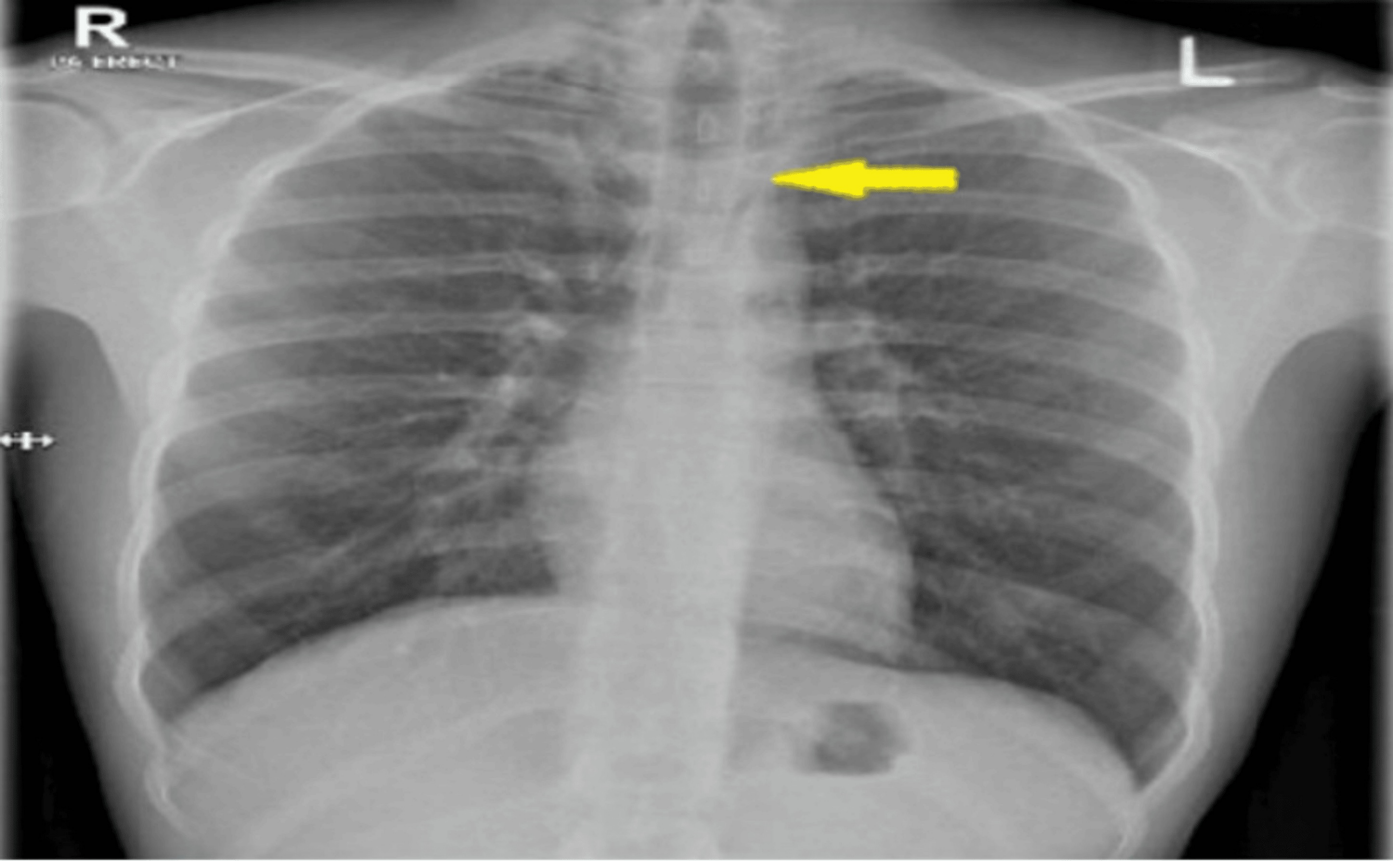
Supine chest X-ray showed air located at the mediastinum (pneumomediastinum) highlighted by the yellow arrow.

A chest X-ray showed no evidence of lobar collapse, pulmonary edema, or pleural effusion. Pneumomediastinum was missed initially (Figure 1).

These findings led the emergency medicine physician and medical team to label the patient as a case of gastritis and admit him to the medical floor. Later that day, the patient started complaining of severe pain with persistent vomiting and mentioned neck pain that did not respond to analgesia.

A gastroenterologist consulted and planned to rule out metabolic causes of vomiting or superior mesenteric syndrome. They asked to repeat and order more investigations (Table 2).

**Table 2:** Additional laboratory test results INR: International normalised ratio; APTT: Activated partial thromboplastin time; PTH: Parathyroid hormone; TSH: Thyroid-stimulating hormone; ACTH: Adrenocorticotropic hormone.

Test	Result	Normal Range
INR	1.3	Critical high >4.9
Prothrombin Time	14.1 seconds	9.4-12.5
APTT	30.4 seconds	25.1-36.5
PTH	30 pg/mL	15-65
TSH	0.42 mIU/L	0.3-4.2
ACTH	16.7 pg/mL ACTH Withdraw Time: PM	
Cortisol	774.0 nmol/L Cortisol Withdraw Time: PM	

The gastroenterologist also recommended good rehydration, antiemetics, proton pump inhibitors as needed, and a computed tomography (CT) scan of the abdomen to rule out obstruction. Before proceeding with the CT scan of the abdomen, the radiology department contacted the assigned physician, mentioned suspicion of pneumomediastinum in the chest X-ray, and asked to order a CT scan of the chest.

**Figure 2:**
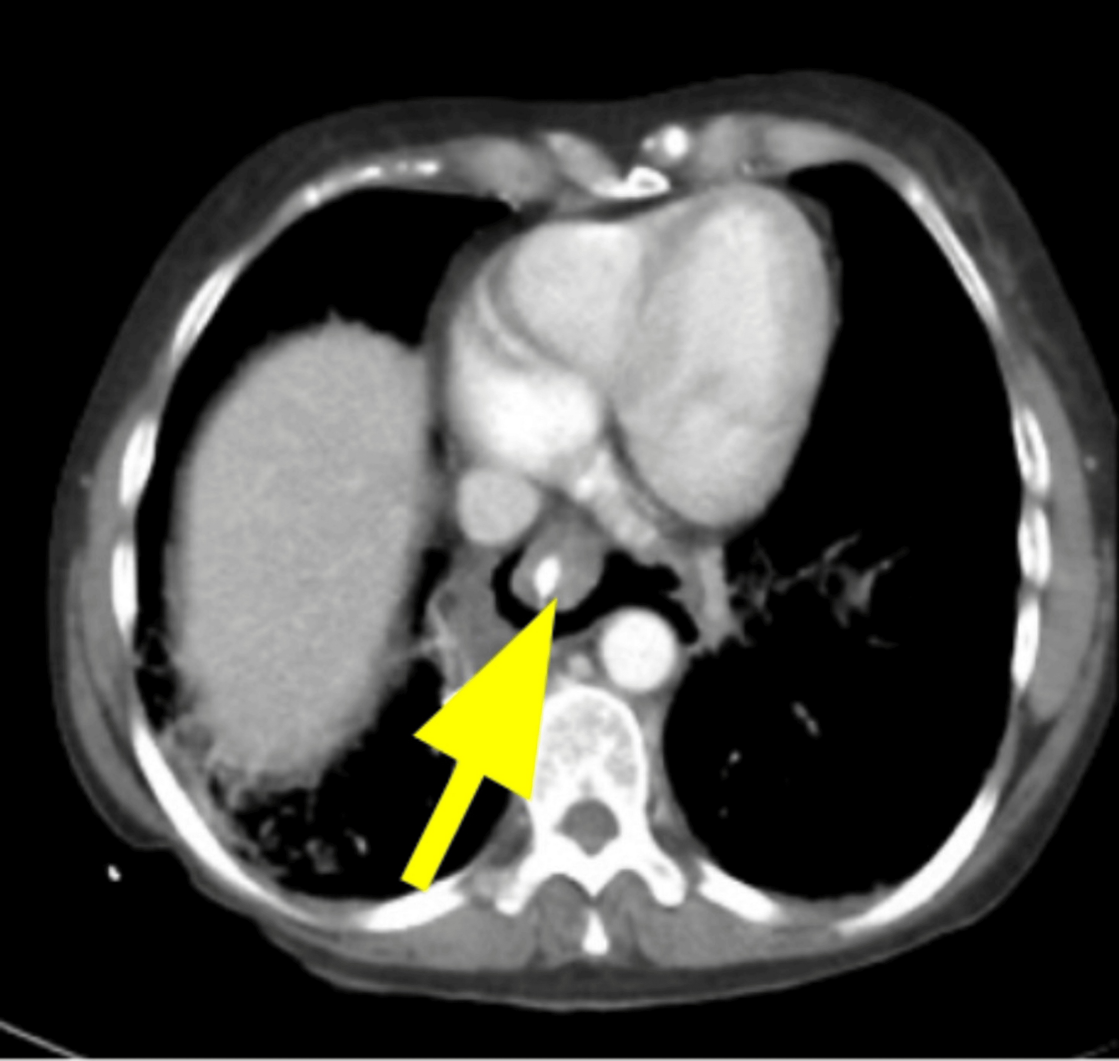
Axial section of CECT chest showed air lobulated area at the mediastinum highlighted by the yellow arrow

The chest CT scan showed esophageal rupture with multiple mediastinal air locules, indicative of pneumomediastinum, extending bilaterally into the neck and the right posterior upper chest wall. Notably, there was no evidence of contrast leak from oral administration. Bilateral lung fields exhibited clearness without consolidation, and the tracheobronchial trees were patent. Additionally, major vascular structures appeared unremarkable, with no pleural effusion observed (Figure 2).

The upper gastrointestinal surgery team was informed, and they advised only conservative management and to keep the patient on total parenteral nutrition (TPN). The patient was also started on enoxaparin 40 mg, subcutaneous injection, daily for three days; metoclopramide 10 mg IV, injection; q8hr PRN as needed for nausea and vomiting; paracetamol PRN for the pain; and potassium chloride 20 mEq + dextrose 5% in 0.9% normal saline 1,000 mL IV.

Upon examination on the day of discharge following a two-week admission, the patient presented well, without distress, exhibiting consciousness, orientation, and alertness. There were no signs of jaundice, pallor, or cyanosis, nor evidence of clubbing, rash, or cervical lymphadenopathy. Abdominal examination revealed a soft and lax abdomen, with no tenderness on deep palpation, guarding, or rigidity, and positive bowel sounds. The patient demonstrated good oral intake and was discharged with a plan for outpatient follow-up in two weeks, along with a regimen of PPI twice daily for the same duration. Recommendations included avoiding spicy foods or any known triggers for gastrointestinal upset and maintaining a pureed diet for an additional four weeks. Discharge medications included PPI and metoclopramide as needed.

## Discussion

Our case represents an 18-year-old man with no past medical history who presented with vomiting and was found to have spontaneous esophageal perforation. Spontaneous esophageal rupture, also known as Boerhaave’s syndrome, is an uncommon condition representing 15% of all causes of esophageal ruptures. It is common in men in their 50s and 60s [5]. However, in our case, the patient was young.

Boerhaave’s syndrome is characterized by a transmural tear of the esophagus, primarily caused by a sudden surge in intraesophageal pressure coupled with negative pressure within the chest cavity while vomiting. Notably, the lower esophageal sphincter functions normally during this process [6]. In Boerhaave’s syndrome, approximately half of the cases present with the classic Mackler’s triad, which includes vomiting, thoracic pain, and subcutaneous emphysema. When dealing with younger patients, it is essential to thoroughly investigate additional risk factors like alcohol consumption. Moreover, exploring other potential causes of vomiting, such as metabolic and surgical factors, is equally important in clinical assessment.

The symptoms of Boerhaave’s syndrome are frequently nonspecific and can overlap with various other medical conditions, including aortic dissection, pancreatitis, myocardial infarction, pulmonary embolism, perforated peptic ulcer, spontaneous pneumothorax, pneumonia, pericarditis, or Mallory-Weiss tear [7]. Distinguishing these disorders from Boerhaave’s syndrome typically involves thorough assessment through history-taking, physical examination, laboratory investigations, electrocardiography, and additional imaging modalities [8].

Nonetheless, esophageal perforation was identified through chest radiography and CT scan. The simple X-ray is a valuable, efficient, and easily transportable diagnostic tool that is particularly beneficial for severely ill patients within closed units [9-11]. In Boerhaave’s syndrome, a notable diagnostic radiological feature is Naclerio’s V sign, observed on chest X-rays as a hyper-lucent V-shaped line [12]. However, this sign is not pathognomonic and may not manifest in lesions at the proximal esophagus level, particularly those of iatrogenic or traumatic origin [9-11]. Additional chest X-ray findings indicative of pneumoperitoneum encompass pneumopericardium, the continuous diaphragm sign, continuous left hemidiaphragm sign, V sign of brachiocephalic vein confluence, and the ring-around-the-aorta sign [9-11].

The timing of patient presentation to the hospital is pivotal in managing esophageal perforation. Initiating treatment within 24 hours of symptom onset significantly enhances patient prognosis, with mortality rates decreasing from approximately 30% to less than 10% [6]. The early presentation of patients and prompt diagnosis play integral roles in facilitating conservative management strategies for esophageal perforation. These approaches become feasible when specific criteria are met, including hemodynamic stability, containment of the esophageal rupture, and minimal mediastinal contamination [13]. This scenario was observed in the management of our patient.

## Conclusions

Boerhaave’s syndrome is uncommon and can often escape early detection, leading to potential complications like dehydration, mediastinitis, sepsis, and shock. Therefore, prompt imaging informs rapid management decisions that are crucial for patient care. Early intervention based on imaging findings minimizes risks and enhances outcomes. Collaborative efforts among specialists ensure comprehensive and effective treatment strategies. Incorporating imaging results into management plans optimizes therapeutic interventions, improving prognosis.
